# Combining Gene Therapy with Current Modulator Treatments for Cystic Fibrosis: A Promising Area of Research

**DOI:** 10.3390/pharmaceutics18081040

**Published:** 2026-08-21

**Authors:** Xavier Buin, Rosy Ghanem, Ines Pankonien, Frédéric Becq, Margarida Amaral, Tristan Montier

**Affiliations:** 1 UnivBrest, Inserm, EFS, UMR 1078, GGB, F-29200 Brest, France; xavier.buin@univ-brest.fr (X.B.); rosy.ghanem@univ-brest.fr (R.G.); 2 Service de Génétique Médicale et de Biologie de la Reproduction, CHU de Brest, F-29200 Brest, France; 3 BioISI—Biosystems & Integrative Sciences Institute, Faculty of Sciences, University of Lisboa, Campo Grande, C8 bdg, 1749-016 Lisboa, Portugal; ipankonien@ciencias.ulisboa.pt (I.P.); msamaral@ciencias.ulisboa.pt (M.A.); 4 Laboratoire PRéTI, University of Poitiers, F-86022 Poitiers, France; frederic.becq@univ-poitiers.fr

**Keywords:** Cystic Fibrosis, CFTR modulators, gene transfer, nucleic acids, combinatory approach

## Abstract

Since the development of the first cystic fibrosis transmembrane conductance regulator (CFTR) modulator in 2012, these therapies have revolutionized patients’ health. They are now the most effective treatment for people with cystic fibrosis (pwCF). In fact, elexacaftor/tezacaftor/ivacaftor and vanzacaftor/tezacaftor/deutivacaftor, the latest combination therapies consisting of a CFTR potentiator and two CFTR correctors, improved lung function by 14% in pwCF. Other modulator therapies targeting CFTR mRNA and/or protein are currently under preclinical/clinical investigation. However, due to the variant-specific nature of these therapies, about 10% of pwCF in Europe remains without effective treatment, and many treated pwCF experience various adverse events such as headaches, infections, hepatotoxicity, hypertension, and depression. Therefore, mutation-agnostic strategies such as gene therapy are needed. They could expand treatment eligibility for all pwCF and improve outcomes. In fact, nucleic acid delivery (e.g., pDNA, mRNA, oligonucleotides, genome editing) or targeting non-CFTR channels to restore ion transport represent promising future additional directions for CF therapy. This review aims to discuss a potential combination between gene therapy approaches and existing modulators to improve treatment eligibility, safety, and efficacy.

## 1. Introduction

### The CFTR Protein and CF-Causing Variants

Cystic fibrosis (CF) is a rare genetic disease caused by variants in the gene encoding the **C**ystic **F**ibrosis **T**ransmembrane conductance **R**egulator (CFTR). The latter is a cyclic AMP-dependent epithelial anion channel [1]. Worldwide, about 188,000 patients are estimated to have CF in 96 countries, with 90,000 people diagnosed, and with the highest prevalence in the European descendant population [2,3].

The CFTR channel is a multidomain membrane protein belonging to the ATP-binding cassette (ABC) transporter family [4]. It is composed of two transmembrane domains (MSD1/2), two nucleotide-binding domains (NBD1/2), and one regulatory domain (RD) [5]. Four conditions are required to enable Cl^−^ efflux through the CFTR channel: (i) phosphorylation of the RD by protein kinase A (PKA) and ATP binding to NBD1, (ii) NBD1-NBD2 dimerization and recruitment of a second ATP molecule, (iii) opening of the channel to allow ion efflux, and (iv) ATP hydrolysis at NBD2, which will lead to the closure of the channel [6]. In 2023, Levring and colleagues proposed a complexified CFTR gating model based on single-molecule resolution [7]. Briefly, once the RD is phosphorylated and ATP is bound to both NBDs without their dimerization, the channel undergoes a series of conformational changes during the gating cycle: (i) NBD dimerization occurs while the channel closed remains closed, (ii) the channel alternates between open and closed configurations while ATP remains bound and NBDs are dimerized, (iii) the channel adopts an open configuration simultaneously with ATP hydrolysis, (iv) a post-hydrolytic closed state in which the NBDs remain dimerized, and (v) a close state with dimerized NBDs and only one ATP bound. From this state, either a second ATP binds to restart at step (ii) or the NBDs dissociate while one ATP remains bound, preparing the channel for the binding of another ATP [7].

**Figure 1:**
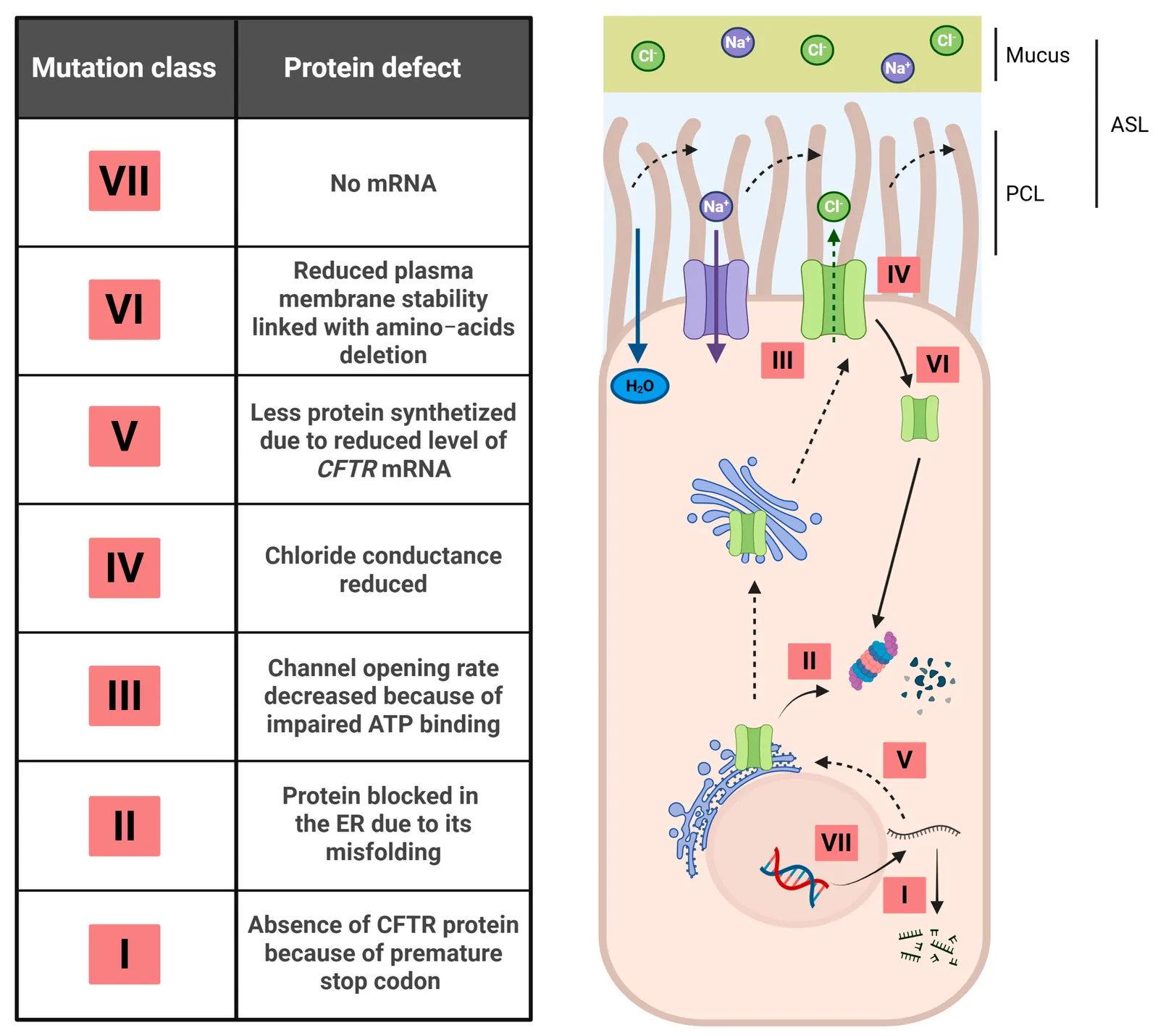
Schematic representation of CFTR variant classes and their impact on CFTR protein and chloride efflux. **Class I** variants refer to frameshift or nonsense variants causing premature stop codons [12]. **Class II** variants are the most represented in pwCF. They cause CFTR protein misfolding, which stays in an immature form and cannot reach the plasma membrane [9]. The p.Phe508del variant is the most common in pwCF. **Class III** variants, or gating variants, impair CFTR protein function [2]. The latter is synthesized and addressed to the plasma membrane but presents diminished functionality due to an alteration of the ATP-binding site. The G551D variant is the most emblematic of this class. **Class IV** variants alter chloride conductance [14]. Those missense variants impact MSD, leading to reduced chloride secretion, although the protein is stable at the plasma membrane (PM). **Class V** variants affect the CFTR pre-mRNA due to alternative splicing and variants in the promoter sequence, leading to reduced levels of CFTR protein at the PM [15]. **Class VI** variants are responsible for the reduction in plasma membrane protein stability. These variants often lead to the deletion of amino acids in the C-terminal reducing the half-life of the protein [10]. **Class VII** variants are called ‘unrescuable’ because of the absence of CFTR mRNA, and thus no protein to be rescued [10]. The main pathway for CFTR mRNA or protein regarding different *CFTR* variants is indicated using solid arrows, whereas dotted arrows represent an alternative pathway leading to low expression of mutated CFTR protein (created with Biorender.com).

Variants in the *CFTR* gene impact either CFTR mRNA stability or CFTR protein processing, stability and/or function [8,9]. Since the identification of the *CFTR* gene, more than 2100 variants have been reported, over 1000 of which result in a CF phenotype (see CF Mutations Database, CFTR2, 2026). Regarding the impact on the CFTR protein, CF-causing variants can be divided into seven classes (Figure 1) [9,10,11,12,13,14,15,16,17]. In fact, the original classification describes six classes: class I includes both nonsense and large deletion variants resulting in no CFTR protein or mRNA being synthesized, respectively. However, a new classification proposes a more precise and accurate framework for distinguishing variants that lead to the production of altered mRNA, which are rescuable (discussed below), and variants that do not allow the production of CFTR mRNA, so-called unrescuable variants [10].

About 80% of people with cystic fibrosis (pwCF) carry a deletion of phenylalanine at position 508 of the protein (p.Phe508del-CFTR, class II), which only residually (~1–5%) reaches the plasma membrane (PM), where it is unstable and shows defective channel gating. The remaining defective protein is being degraded by endoplasmic reticulum-associated degradation [2,11,18,19].

Recent advances were made in the past decade, such as the development of modulator-based therapies and upgrades of various gene delivery approaches that are currently in clinical trials (CTs). In fact, approved CFTR modulators, which are small molecules acting on the CFTR protein, result in about 14% improvements in lung function, thus strongly increasing pwCF quality of life and life expectancy [20]. Although this approach is available for most pwCF aged at least 2 years old, it is not suitable for all patients and shows variable efficacy. Therefore, in the future, gene therapy could fill this gap by providing mutation-agnostic treatment for CF.

In this review, we aim to describe strategies and current treatments for CF, focusing primarily on gene therapy approaches and modulators, and highlighting their respective limitations and perspectives. Subsequently, we highlight published evidence suggesting the possibility of additional or synergistic effects while combining the two approaches.

## 2. The Re-Emergence of Gene Delivery for CF

Gene delivery has represented a major therapeutic option since the discovery of the *CFTR* gene in 1989, largely because of its variant-agnostic nature. Historically, gene therapy was the first strategy explored to treat pwCF [21,22,23,24]. Two main types of vectors can be used for nucleic acid (NA) transfer: viral and non-viral vectors. Unfortunately, for CF, none of them showed significant improvement in pwCF pulmonary function, and no treatment has yet been clinically approved, mainly due to limited penetration through the thick mucus and, consequently, low expression levels [25]. In fact, various studies indicate that restoring about 10% of wild-type (wt) CFTR function is required to observe a clinical benefit, which was never reached during gene transfer CTs [26,27,28]. Nowadays, various gene therapy approaches are under investigation for CF treatment, including DNA or mRNA delivery, genome editing, and others. Most CTs of CF gene therapy have used aerosol delivery to directly target the lungs of pwCF. However, several challenges need to be overcome to potentially use this strategy. The vector must (i) be suitable and optimized for the administration method; (ii) protect the NA sequences from degradation; (iii) be able to penetrate the thick mucus layer; (iv) enable efficient expression in the targeted airway cells; and (v) not induce an immune response to allow repeated administration depending on the gene transfer approach used.

### 2.1. Viral Gene Delivery

**Figure 2:**
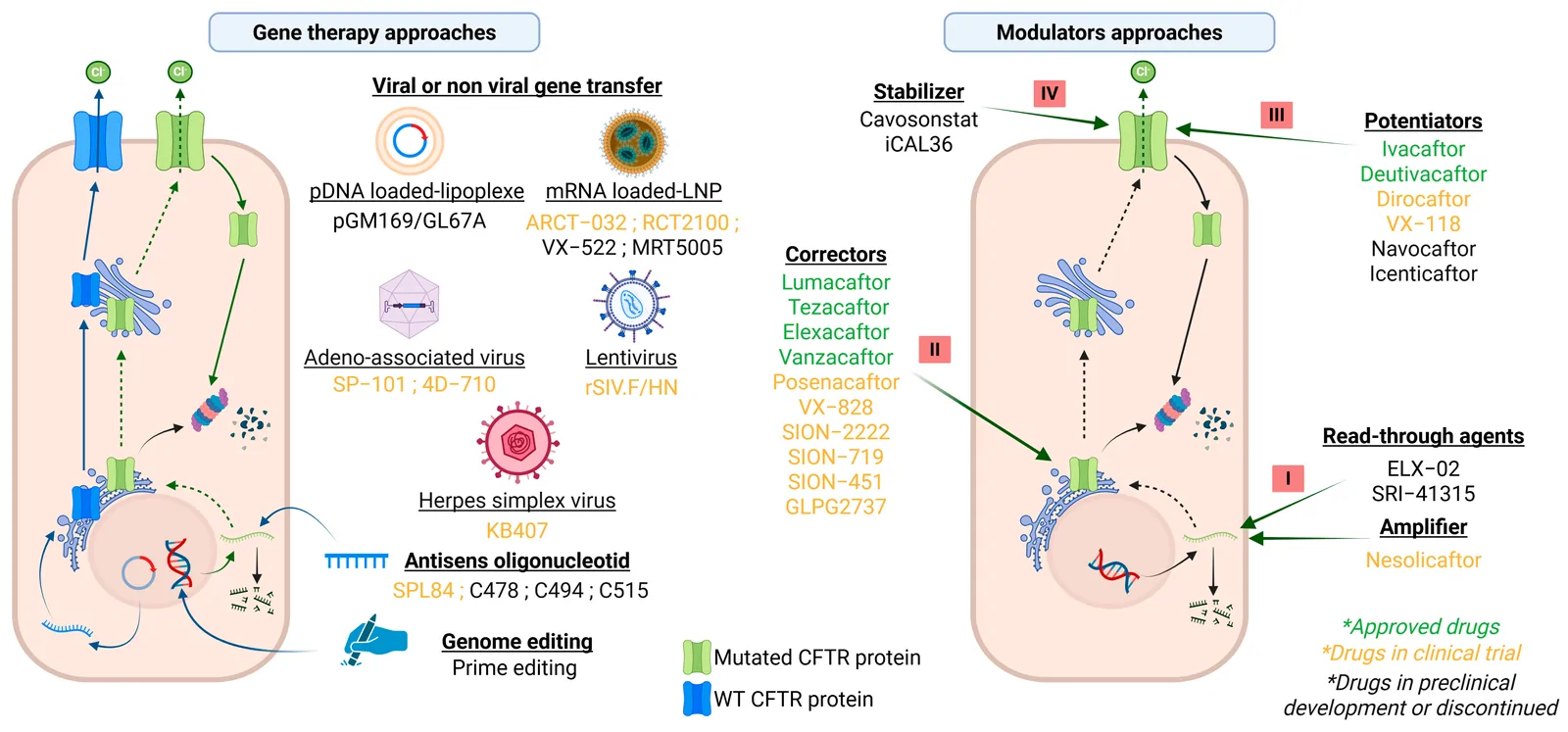
Schematic representation of various gene therapy approaches, and CFTR modulators approved (green), being evaluated in CT (orange) or under preclinical development or discontinued (black) for CF. **In-development** **gene therapy approaches:** Viral and non-viral vectors are used to deliver NAs (pDNA or mRNA) encoding a functional CFTR protein. Depending on the vector, the exogenous gene may integrate into the host genome or not, resulting in either permanent or transient expression, respectively. Genome editing strategies aim to permanently correct the genome sequence, while ASOs rely on RNA base pairing to modulate abnormal mRNA splicing (and PTCs in some cases). **Approved CFTR modulator approaches:** Correctors improve CFTR protein folding, maturation, and trafficking to the plasma membrane (PM) and are primarily designed for class II variants. Potentiators increase the CFTR channel opening probability at the PM. **CFTR modulators in development:** Amplifiers are molecules that stabilize CFTR mRNA by increasing its half-life, thereby enhancing CFTR protein synthesis. Read-through agents promote insertion of a foreign amino acid at PTCs during CFTR mRNA translation and are being developed for class I variants (created with Biorender.com).

Historically, adenoviral (Ad) vectors encoding the full-length human *hCFTR* cDNA were used in the first gene therapy CT for CF [21]. However, Ad vectors’ efficiency and safety differed in CT from in vitro results [22]. In fact, they induced airway inflammation, and neither *CFTR* mRNA nor CFTR protein from the transgene was detected during the application in CTs [22]. Subsequently, other viral vectors, such as adeno-associated viruses (AAVs), have been developed, as they have a higher tropism for airway epithelial cells and are less immunogenic than Ad vectors. As an example, in Ad-based trials, AAV CTs demonstrated a safety profile, but no clinical benefit was observed between the AAV and placebo groups for CF [29]. However, AAV vectors are already used in gene therapy for other diseases such as inherited retinal dystrophy [30]. Moreover, recent developments and the identification of new AAV variants have enabled improvements in AAV-based approaches. Currently, an ongoing phase I/II CT is evaluating the aerosol delivery of an optimized recombinant AAV (AAV2.5T) with high tropism for lung epithelial cells, especially their apical surface, and carrying the SP183 enhancer promoter sequence, SP-101, instead of the usual (Figure 2) (NCT06526923) [31]. The latter aims to express *hCFTRΔR* in people who are ineligible or intolerant to CFTR modulator therapies [31]. Due to the limited capacity of AAV (no more than 4.7 kb) to carry long NA sequences and to improve gene expression using AAV, the RD of CFTR was shortened (*hCFTRΔR*), allowing the inclusion of a strong promoter and resulting in the expression of a functional CFTR channel [22]. Another aerosol-administered AAV-based gene therapy, using an AAV variant A101 and containing a codon-optimized and *hCFTRΔR* sequence (4D-710), is currently enrolling participants for its phase I/II CT [32,33,34] (NCT05248230).

Lentiviral vectors represent another type of delivery system capable of transducing non-dividing cells with a low immunogenicity level. These vectors, derived from viruses such as human immunodeficiency virus (HIV), feline immunodeficiency virus (FIV), equine infectious anemia virus (EIAV), and simian immunodeficiency virus (SIV), are capable of integrating into the host genome [35]. In contrast to AAV vectors, lentiviral vectors can package the full-length *hCFTR* cassette and have been investigated for CF since the 1990s, owing to their ability to deliver their cargo to the airways of various CF animal models. In fact, their tropism can be further optimized for lung epithelial cells thanks to their glycoprotein envelope. Interestingly, rSIV.F/HN, a hybrid lentiviral vector based on an SIV pseudotyped with Sendai virus envelope proteins F and HN, has been shown to enhance airway epithelium transduction efficiency in preclinical studies [36]. This vector showed promising results in restoring CFTR-mediated currents in air–liquid interface (ALI) cultures of primary human bronchial epithelial (HBE) cells from pwCF, and the combination with ivacaftor further enhanced CFTR function [37]. Based on these findings, the first CT of a lentivirus-based gene therapy for inhaled BI 3720931 (rSIV.F/HN) is currently ongoing (NCT06515002) (Figure 2) [17].

Another phase I CT is enrolling participants for repeated nebulization of KB407, a herpes simplex virus type 1 (HSV-1)-based gene therapy (Figure 2). The latter harbors a natural tropism for epithelial cells and enables the expression of full-length CFTR protein in both healthy and pwCF-derived HBE cells (NCT05504837) [38].

### 2.2. Non-Viral Gene Delivery

Numerous gene therapy CTs for CF using non-viral vectors aim to deliver a wt copy of the *hCFTR* pDNA or mRNA. The last pDNA-based trial was published in 2015 by Alton et al. [39]. They used a CpG-free plasmid encoding an optimized *hCFTR* gene (pGM169) formulated with GL67A, a lipid mixture composed of the cationic lipid GL67 (derived from spermine), dioleoylphosphatidylethanolamine (DOPE), and DMPE-PEG5000 [40] (Figure 2). Despite encouraging outcomes in in vivo models, repeated nebulization of pGM169/GL67A lipoplexes only led to the maintenance of ppFEV_1_ in pwCF. These results are likely due to the viscous mucus that traps nanoparticles, highlighting the need for further optimization of this approach. Nevertheless, the treatment was well tolerated, with no side effects reported even after multiple administrations [39,40].

Two CTs are currently ongoing for mRNA-based delivery, all employing lipid nanoparticles (LNPs), a more recent type of synthetic vector. LNPs are composed of an ionizable cationic lipid, a helper lipid such as 1,2-distearoyl-*sn*-glycero-3-phosphocholine, cholesterol, and polyethylene glycol (PEG). LNPs can efficiently encapsulate and deliver mRNA when administered via aerosol [41]. Briefly, this strategy aims to deliver mRNA encoding a wt CFTR protein, based on the ability of LNPs to deliver nucleic acid into the cytosol after endosomal uptake and escape mediated by the ionizable lipid [42]. Thus, mRNA can be translated, allowing a transient expression of functional CFTR proteins.

Currently, a phase II study is enrolling participants to receive ARCT-032 via nebulization, which is composed of *hCFTR*-mRNA encapsulated in LNPs (NCT06747858) (Figure 2). Preclinical studies with ARCT-032 revealed efficient restoration of CFTR activity in human airway CF cell models, CF mice, and CF ferrets. Furthermore, the phase I CT demonstrated that ARCT-032 was safe and well tolerated [43]. A second inhaled mRNA-based therapy, RCT2100, is currently under evaluation in a Phase I/II trial (Figure 2) (NCT06237335). RCT2100 delivers full-length *hCFTR* mRNA encapsulated in selective organ targeting (SORT) LNPs, which are designed to specifically target tissues, such as the lungs [44]. Recently, the early termination of the CT evaluating VX-522, an inhaled LNP-mRNA therapy, was announced, precluding an assessment of the safety and efficacy of this compound (Figure 2) (NCT05668741). Moreover, another phase I/II CT was conducted, and the results were published in 2023 (NCT03375047) [45]. This study aimed to evaluate the safety and efficacy of MRT5005, a codon-optimized *hCFTR* mRNA delivered as an LNP-formulated aerosol in pwCF with two severe class I or II variants. Unfortunately, even if MRT5005 was generally safe and well tolerated after multiple nebulizations, pwCF FEV_1_ remained stable after the treatment compared with the placebo group. For now, this compound is no longer evaluated in CT [45]. In fact, an mRNA-based approach could enable only a transient expression of CFTR protein and thus require multiple administrations. Moreover, synthesized mRNA requires modifications such as methylation or capping modifications to improve mRNA stability [46,47]. Finally, exogenous mRNA may trigger an immune response or be degraded, thus reducing transfection efficiency [48].

### 2.3. Development of Antisense Oligonucleotides and Genome Editing for CF

In addition to variant-agnostic strategies, variant-specific approaches using gene therapy are under development. First, synthetic chemically modified antisense oligonucleotides (ASOs) are RNA-like molecules that modulate pre-mRNA splicing or translation by base pairing with target mRNA sequences [49]. Thus, they can counteract CF splicing variants (and some nonsense or premature termination codons (PTCs)), which often lead to mRNA degradation via the nonsense-mediated RNA decay (NMD) or to the production of a truncated or non-functional CFTR protein [50]. In some cases, CFTR modulators have been able to rescue misfolded proteins resulting from PTCs located towards the end of the CFTR protein [51]. For now, SPL84 is the only ASO candidate currently evaluated in CT to rescue the 3849 + 10 kb C->T splicing variant. Previous studies in HBE cells from pwCF with this variant demonstrated correction and restoration of CFTR activity [52,53]. A study is currently enrolling participants in a phase I/II CT for inhaled SPL84 (NCT06429176). Preliminary results have shown that SPL84 was safe, well tolerated, and did not induce significant adverse effects, enabling the beginning of phase II [54]. Other ASOs are under preclinical development for CF. For example, a recent study demonstrated that a cocktail of ASOs decreased NMD activity in cells harboring the Trp1282X variant [49,55]. This was accomplished by three ASOs (C478/C494/C515) that inhibit the interaction between the PTC-containing exon and the exon junction complex, which is responsible for the recruitment of NMD components [55]. For now, this approach is still in preclinical development with in vivo models. Other ASOs inhibiting components of the NMD pathway were studied by Sanderlin and colleagues [50]. They identified SMG6 as a crucial component of NMD for mRNA degradation. Briefly, this ASO binds SMG6, forming a DNA-RNA heteroduplex that is targeted for degradation by the ubiquitous endonuclease RNase H, therefore reducing SMG6 expression [56].

Beyond ASOs, genome editing, including CRISPR-based and CRISPR-derived approaches, represents another promising avenue for CF therapy (Figure 2) [57,58]. CRISPR-based genome editing relies on the Cas9 nuclease, guided by an RNA sequence that recognizes the target DNA, inducing a double-strand break. This break can subsequently be repaired by homologous recombination in the presence of a DNA donor template. However, this approach requires further optimization to address risks such as off-target effects and potential DNA damage resulting from double-strand breaks [9]. On the other hand, CRISPR-derived genome editing, such as prime editing, offers a potentially safer alternative. It uses a fusion protein that combines Cas9 with an engineered reverse transcriptase, which directly synthesizes DNA from the prime editing guide RNA (pegRNA) into the target genomic *locus*. Unlike conventional CRISPR-based editing, prime editing does not generate double-strand breaks, thereby reducing the risk of off-targets and DNA damage [59,60]. Preclinical studies delivering the prime editing components by electroporation into patient-derived intestinal organoids (PDIOs) with nonsense or p.Phe508del variants demonstrated the restoration of CFTR function [60]. Moreover, a CRISPR-based approach delivered by lung-targeted SORT LNPs enabled homology-directed repair in homozygous G542X mice and in human bronchial epithelial cells derived from homozygous p.Phe508del donors, thus improving CFTR-dependent chloride efflux [61]. These results suggest that with further development, genome editing could be applied efficiently to treat CF.

### 2.4. Challenges for Gene Therapy in CF

As mentioned before, no gene therapy approach is available for CF, and most CTs have resulted only in maintenance of lung function in pwCF rather than its improvement. Multiple factors can explain this data.

First, most CTs have tried to deliver gene therapy agents by nebulization. However, the thick mucus layer is one of the main biological barriers as it traps nanoparticles due to its negative charges and the dense meshwork created by components such as extracellular DNA and non-expanded mucins [39,62,63,64]. Therefore, the size of gene therapy nanoparticles and their ability to resist shear forces generated during the aerosolization process are limiting factors [64,65].

Furthermore, various studies highlight the need to improve cell targeting to enhance transfection/transduction efficiency. For example, pulmonary ionocytes that are responsible for more than 60% of CFTR-mediated chloride efflux in healthy subjects, and basal cells, which are responsible for airway epithelium renewal, represent promising targets for restoring CFTR activity [66,67,68,69]. Notably, previous CT did not specifically target specific cells in the epithelium. This way, new viral vector serotypes need to be further identified, and lung-targeted SORT LNP (apical or basal pole depending on the administration technique) should be further optimized.

Moreover, vectors capable of efficiently transfecting/transducing both dividing and non-dividing cells with long-term expression could improve the gene therapy approach, as pulmonary airway epithelial cells are mostly non-dividing with a forty-day turnover. For example, current CTs with synthetic vectors are using mRNA instead of pDNA, which was used in previous CTs. In fact, unlike mRNA, pDNA requires nuclear import, a process facilitated by the dismantling of the nuclear envelope during mitosis [70]. Therefore, mRNA could be more efficient for transfecting non-dividing cells. As mentioned before, the choice of gene delivery vehicle imposes specific constraints. The limited encapsulation capacity of AAV vectors may require adapting the NA sequence, while repeated administration may be necessary depending on the duration of transgene expression and epithelial cell renewal [31,32].

In fact, permanent expression of wt CFTR proteins would require a strategy such as integrative virus or genome editing and would also require targeting basal cells. Thus, most approaches would require frequent re-administration, due to the half-life of pDNA or mRNA and cell renewal. For example, CT delivering pDNA using non-viral vectors only enabled transient expression lasting about four weeks [22]. Preclinical studies show that AAV2.5T-mediated CFTRΔR expression decreases after 10 days in adult ferrets [71]. However, interim results from 4D-710 CT seem to show a durable expression of transgene CFTR mRNA after one dose [72]. Moreover, during the CT evaluating MRT5005, a study group received a single dose of mRNA. For these patients, lung function slightly improved but returned to placebo levels within a few days [45].

However, repeated administrations require that gene delivery does not induce inflammation or an immune responses that could reduce gene transfer efficacy. Nevertheless, several CTs, especially those involving viral vectors, have been shown to induce an immune response following repeated administrations or because of pre-existing antibodies recognizing the virus capsid [73,74,75]. To overcome these limits, efforts are made to develop novel capsids that resist pre-existing antibodies, such as the capsid used in the CT evaluating 4D-710 [34].

For now, CTs using non-viral vectors have led to the best safety profile. Moreover, most CTs for gene transfer in CF have been undertaken in adults with CF, as most already have advanced lung disease. Therefore, developing approaches that could be used early in patients’ lives to prevent irreversible damage from disease progression, as tested for other diseases like Duchenne muscular dystrophy, should also be considered [76]. Indeed, with the development of gene-editing technologies, many studies are exploring in utero gene therapy with various models to treat the entire organism rather than targeting only the lungs [77,78]. However, gene therapy in children and at early life raises multiple issues such as considerations of growth, long-term safety, and ethical dilemmas [78,79].

Above all these weaknesses, most of the mentioned CTs demonstrated that gene therapy is generally safe and well tolerated. Thus, it still holds great potential for CF, by its ability to provide agnostic treatment to all pwCF.

## 3. CFTR Modulators: A Clinical Milestone for CF Treatment

In parallel with gene therapy research, the development of drugs directly targeting CFTR protein defects has become a major focus of research. For this purpose, multiple orally administered drugs have been developed and evaluated in CT since the beginning of 2010. Overall, these treatments improved pwCF lung function, thus contributing to an increased life expectancy, with a median predicted survival age of over 50 years, depending on when patients were born [20,80,81,82,83,84,85]. These kinds of molecules, called CFTR modulators, operate at different levels, from CFTR mRNA to membrane-addressed protein, aiming to restore a wt CFTR phenotype in pwCF [2,9]. Approved CFTR modulators fall into two categories: potentiators and correctors [9]. In 2017, Jue Chen and colleagues resolved the human CFTR protein structure thanks to high-resolution electron cryo-microscopy (cryo-EM). This work provided key insights into the mechanism of action of both correctors and potentiators [7,86,87,88]. Nevertheless, the CFTR structure established with cryo-EM lacks the R domain, and the CFTR structure in a channel-opened configuration has never been resolved. Thus, further progress is still needed to better understand the relationship between CFTR conformation and function.

Additional modulator classes are under development (see Cystic Fibrosis Foundation Drug Development Pipeline, https://apps.cff.org/trials/pipeline, accessed on 27 July 2026). To date, all clinically approved CFTR modulator drugs have been identified by high-throughput screening (HTS) combined with medicinal chemistry (Table 1 and Table 2), and many other drugs are still under development by various companies [89,90,91].

### 3.1. Ways to Improve CFTR-Mediated Chloride Efflux: CFTR Activators and Potentiators

CFTR modulators classified as potentiators and activators enhance CFTR channel activity and thereby restore Cl^−^ efflux in mutated cells [2,92]. For instance, potentiators increase the channel-opening probability, whereas activators raise cAMP levels [92]. Moreover, potentiators’ binding site within the CFTR protein may differ depending on the molecule. Therefore, the gain of CFTR function is mediated through different mechanisms.

Potentiators and activators were first developed for class III and IV variants (Figure 1). However, potentiators are now widely used in combination with correctors for other variants such as p.Phe508del (class II) [20,83,85,91,93,94]. In fact, numerous activators failed to demonstrate a real benefit during CT (see Cystic Fibrosis Foundation Drug Development Pipeline, https://apps.cff.org/trials/pipeline, accessed on 27 July 2026). As an example, the activator genistein indirectly improves CFTR channel activity in vitro by increasing cAMP levels through the inhibition of protein-tyrosine kinase (Table 1) [95]. However, this molecule did not demonstrate any clinical efficacy in pwCF during CT, and its development has now been discontinued (NCT00590538). To date, no CFTR activator is marketed.

**Figure 3:**
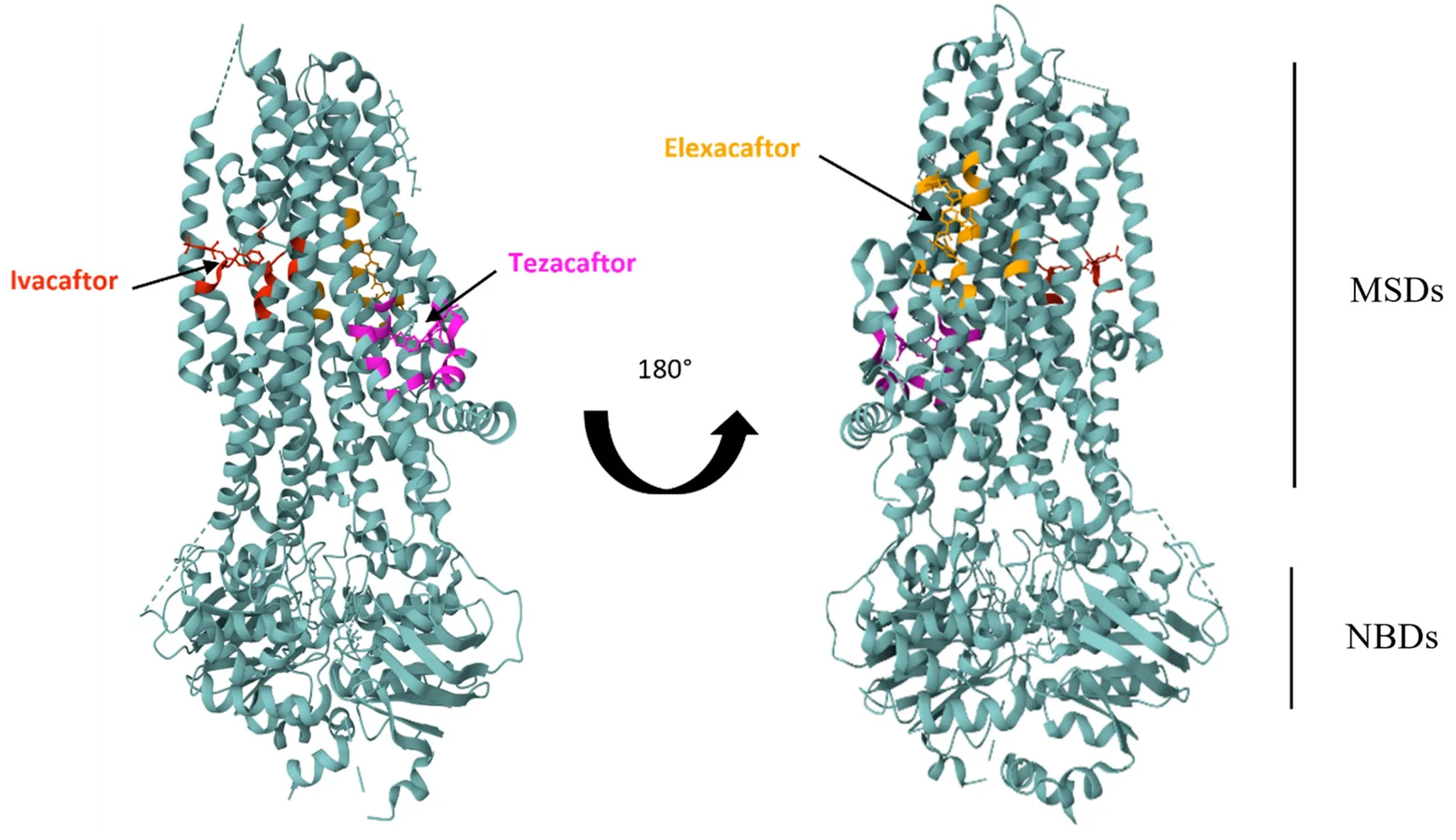
Three-dimensional representation of phosphorylated human p.Phe508del-CFTR with elexacaftor/tezacaftor/ivacaftor and ATP/Mg based on K. Fiedorczuk and J. Chen’s work (Protein Data Bank ID: 8EIQ, adapted from www.rcsb.org/). Clinically FDA/EMA-approved CFTR modulators represented are ivacaftor (potentiator), tezacaftor and elexacaftor (correctors), and their binding sites. **Ivacaftor** (red) binds in an MSD2 pocket formed by TM helices 4, 5 and 8. **Tezacaftor** (purple) fills a pocket by interacting with TM helices 1, 2, 3 and 6 in MSD1. **Elexacaftor** (orange) also binds an MSD2/MSD1 cavity, interacting with TM helices 10 and 11 (MSD2), and 2 (MSD1) [88]. CFTR modulators and their binding sites are represented in colors.

On the other hand, many potentiators are currently approved or evaluated in CT for CF. Ivacaftor (VX-770) is a CFTR potentiator that binds directly to the CFTR protein and has proven its clinical efficacy (Table 2) [86,96,97]. It was the first modulator clinically approved for class III variants such as p.Gly551Asp [84,86,91]. Nevertheless, ivacaftor interacts via hydrogen bonds with the CFTR protein [92]. A first binding site was described by Liu et al. using cryo-EM and a mutated CFTR protein, p.Glu1371Gln (E1371Q) CFTR. These authors revealed that ivacaftor interacts with p.Glu1371Gln-CFTR at MSD2 and the protein–lipid interface by fitting into a cleft formed by TM helices 4, 5, and 8 (Table 1 and Figure 3) [86]. Later, a second binding site was proposed by Laselva et al., supported by data obtained in microsomal membranes and therefore without a cryo-EM study [96]. This second binding site seems located in the intracellular loop 4 (ICL4) at the NBD1/MSD2 interface, and it is less accepted by the scientific community [96]. Studies using specific variants in ivacaftor binding sites of wt CFTR protein have shown that both motifs are critical for ivacaftor acting on CFTR protein [98]. In fact, in ivacaftor-bound p.Glu1371Gln-CFTR (without any variant on its binding sites), the channel-opening probability is increased without promoting NBD dimerization [7]. Ivacaftor seems to mimic the allosteric signaling pathway normally triggered by NBD dimerization, thereby increasing the channel open probability without promoting NBD dimerization when the R domain is phosphorylated [99]. Deutivacaftor, a deuterated analogue of ivacaftor with improved pharmacokinetics, is the latest next-generation potentiator (Table 1) [85]. In a phase II CT comparing deutivacaftor monotherapy (one dose per day) versus ivacaftor monotherapy (two doses per day) in pwCF with gating variants, preliminary results revealed an increase in lung function [85]. Thus, deutivacaftor is now clinically approved and part of the latest triple combination modulator-based drug released on the market for CF treatment (Table 2 and Figure 2) [85].

Elexacaftor (VX-445) is another CFTR modulator, primarily known for its corrector effect (will be discussed below), that also exerts a potentiator effect on p.Phe508del, Gly551Asp and wt CFTR [97,100,101]. In fact, in various electrophysiological experiments, acute addition of elexacaftor increases CFTR-mediated chloride efflux in wt and mutated cells [97,100]. This effect seems complementary to ivacaftor, suggesting a different mechanism of action, whereas elexacaftor directly interacts with the CFTR protein [88,97,100]. Indeed, elexacaftor directly binds TM helix 2 (MSD1) and TM helix 10 (MSD2) and forms electrostatic and van der Waals interactions with TM helix 11 (MSD2), thus enabling NBD1 stabilization (Figure 3) [88]. Since 2024, a third-generation CFTR potentiator, VX-118, has been under clinical development. A first CT evaluated VX-118 bioavailability, and nowadays, the safety and tolerability of this compound are evaluated in triple combination with tezacaftor and VX-828 (a CFTR corrector) (Table 1) (NCT06312787, NCT06154447). Based on current potentiator binding sites, some authors propose a classification of these molecules into different types, depending on their binding site within the CFTR protein [102]. For now, this classification is not very widely used by the scientific community.

Above these marketed drugs, various potentiators are currently in CT, such as dirocaftor (PTI-808). The latter is evaluated in association with posenacaftor (a CFTR corrector) and nesolicaftor (a CFTR amplifier) in pwCF harboring rare CF-causing variants that are not eligible for current CFTR modulator treatments (Table 1) (NCT06468527). Dirocaftor is chemically similar to ivacaftor and increases the CFTR channel-opening probability, although the precise underlying mechanism remains unknown [103,104]. This CT is conducted in two phases. First, intestinal organoids from pwCF were tested to predict the clinical drug response. Therefore, the three CFTR modulators are evaluated in selected pwCF. Preliminary results showed that the combination of these three drugs was well tolerated, with mostly mild to moderate reversible side effects [105]. Navocaftor (SION-3067), developed by Sionna Therapeutics^®^ (Waltham, MA, USA), is another potentiator that was evaluated in CT in association with galicaftor (ABBV-2222, a CFTR corrector) (Table 1) (NCT04853368). This molecule interacts with CFTR MSD2 and exerts the same biological activity as ivacaftor, without any additive effect when combined with the latter [106]. However, CT did not meet the expected results, and the development of this molecule has now been discontinued (NCT04853368).

Icenticaftor (QBW251) is another CFTR potentiator evaluated for both CF and chronic obstructive pulmonary disease (COPD) [104,107]. This molecule was recently evaluated in phase I/II CT on healthy volunteers, a few pwCF harboring class III and IV variants or homozygous p.Phe508del, and demonstrated a safety profile (Table 1) (NCT02190604) [108]. Improvement of lung function was observed only in patients harboring a class III or IV variant [108]. However, no further CT is planned with this molecule regarding CF. Nevertheless, a recent study published by D.M. Cholon and colleagues in 2025 comparing icenticaftor and ivacaftor suggests that icenticaftor is a better option than ivacaftor, as improving elexacaftor/tezacaftor rescued p.Phe508del CFTR-mediated chloride efflux [109]. In fact, no destabilization of rescued p.Phe508del was observed with icenticaftor, in contrast to ivacaftor, as previously described [109,110]. However, a recent study suggests that in vitro ivacaftor-mediated destabilization of rescued p.Phe508del CFTR depends on cell culture conditions [111]. Therefore, more studies are required to identify the precise impact of ivacaftor on rescued p.Phe508del CFTR. Unlike for CF, icenticaftor was recently evaluated in CT for COPD, and recent results showed a small improvement in patients’ lung function at 24 weeks [107].

### 3.2. CFTR Correctors: Rescuing the CFTR Protein

CFTR correctors have been developed to correct the folding and PM trafficking defects of misfolded CFTR protein, such as those generated by the p.Phe508del-CFTR variant, preventing its rapid degradation. These compounds directly increase the amount of CFTR protein by improving its folding through direct interaction with the protein. Historically, correctors have been classified into three types (I–III) regarding their additivity and their respective CFTR binding sites. However, this organization is in constant evolution due to the development of new modulators and the complexity of their features and mechanisms of action [112]. For example, since 2024, a fourth class of correctors has been proposed [112,113]. Moreover, some modulators in development are commonly called NBD stabilizers although they are CFTR correctors. Thus, they do not belong to historical type I-III correctors and have different mechanisms of action. In fact, correctors should be precisely classified according to their mechanisms of action to better characterize their distinct properties and identify rational combination strategies. Overall, the majority of CFTR modulator mechanisms of action have been elucidated only recently [88].

Lumacaftor (VX-809, type I) was the first approved corrector for pwCF either homozygous or heterozygous for p.Phe508del variant (Table 1 and Figure 2) [114]. The combination with ivacaftor led to a modest ppFEV_1_ improvement (~3%) and a reduction in pulmonary exacerbations (Table 2) [83,114]. In fact, lumacaftor increases p.Phe508del-CFTR cell surface expression by improving protein folding, which was also observed for various other CFTR mutants and for wt-CFTR protein [115,116]. This pharmacological chaperone also increases p.Phe508del-CFTR stability at the PM [87]. Lumacaftor binds directly within a deep pocket formed by TM helices 1, 2, 3 and 6 in MSD1 at the protein/lipid interface via van der Waals interactions independent of NBD dimerization [87]. Tezacaftor (VX-661), another type I corrector, is also administered in combination with ivacaftor in pwCF with two p.Phe508del-CFTR variants or just one plus residual function (RF) variant (Table 2) [117,118]. As it is chemically similar to lumacaftor, tezacaftor binds the same hydrophobic pocket in MSD1 by filling the internal cavity but interacts with fewer residues (Table 1 and Figure 3). Both small molecules stabilize the MSD1/NBD1 interface during translation and folding, thus promoting p.Phe508del-CFTR PM trafficking and preventing its degradation [119]. Recently, a combination of deutivacaftor, vanzacaftor (a new CFTR corrector) and tezacaftor was approved for pwCF with at least one p.Phe508del variant or one non-class I variant [85].

Elexacaftor (VX-445) is historically a type III corrector (Figure 2). It is commercialized in association with both tezacaftor and ivacaftor (which was the first triple drug released on the market for CF), resulting in a 10–13.8% improvement in pwCF ppFEV_1_ eligible for this treatment (Table 2) [20,120,121]. This treatment is available for pwCF with at least one p.Phe508del-CFTR variant and was extended by the FDA and EMA for 271 responsive CF-causing variants following in vitro studies [122,123,124]. As mentioned above, elexacaftor directly binds the CFTR protein, enabling NBD1 stabilization, and acts synergistically with tezacaftor to improve the folding of p.Phe508del-CFTR through direct contact, electrostatic, and van der Waals interactions in MSD1 (Table 1) [88]. While the elexacaftor binding site is known, its precise mechanism of action remains unclear. Various studies indicate a direct stabilization of NBD1, whereas others suggest that elexacaftor improves domain assembly without stabilizing NBD1 [88,122,125,126]. Potentiator and corrector roles of elexacaftor are accomplished via the same binding site, which could also enhance the channel function of wt-CFTR and various CFTR mutants [84,88,91]. Elexacaftor is a good example illustrating the complexity of CFTR modulator classification because of its dual effect on CFTR protein. Another type III corrector in CT—posenacaftor (PTI-801)—is described as sharing the same mechanism of action as elexacaftor (Table 1) (NCT06468527) [127]. Vanzacaftor (VX-121) is the latest corrector released on the market. This type III corrector improves p.Phe508del CFTR folding, resulting in an increased p.Phe508del CFTR amount at the PM (Table 1). It is used in association with deutivacaftor and tezacaftor (Table 2) [85]. For now, the mechanism of action of vanzacaftor remains unknown. However, this molecule replaces elexacaftor in the triple combination therapy.

In addition to those approved correctors, many were or are evaluated in preclinical or clinical studies. As mentioned before, a fourth type of corrector has been proposed recently by Marchesin et al., consisting of a small molecule that binds the lasso helix 1 in MSD1 (Table 1) [113]. This molecule increases folding of wt, p.Phe508del, and other CFTR mutants and acts additively with type I, II, and III correctors [113]. Other types of correctors are investigated to improve p.Phe508del-CFTR protein folding. First, VX-828, a third-generation CFTR corrector, is currently in CT for pwCF and healthy participants (Table 1) (NCT06154447). Moreover, type II correctors, such as C4 and core-corr-II, are examples of molecules that interact with NBD2 to improve protein folding (Table 1) [112]. For now, no type II corrector has been clinically approved due to limited efficacy. However, a recent type II corrector, named GLPG2737, has been identified (Table 1) [128,129]. This corrector improves p.Phe508del maturation and trafficking alone and while used in combination with a type I corrector such as lumacaftor [128]. However, comparison of chloride efflux in CF cells from donors treated with GLPG2737 alone or in triple combination showed a slight decrease in channel function when this modulator was not combined with a potentiator [129]. Currently, the development of this compound has been discontinued regarding CF [129].

Galicaftor (SION-2222) is another corrector under investigation in a phase I CT (Table 1) (NCT07035990). This type I corrector has a similar structure to lumacaftor and tezacaftor and also targets the MSD1 of the CFTR protein [104,130,131]. This compound was evaluated in multiple CTs as a triple combination with either Navocaftor/ABBV-119 or Navocaftor/ABBV-567 (NCT04853368, NCT05538585, NCT03969888, NCT03045523, NCT03119649). Overall, galicaftor was well tolerated, whereas no significant clinical improvement was demonstrated [132]. Currently, this compound is undergoing a phase I CT in combination with two correctors, SION-451 and SION-109, to evaluate the safety and potency of these compounds in healthy subjects (NCT07035990). Moreover, SION-109 targets the ICL4 region of the CFTR protein [133].

Other correctors aim to stabilize the NBD1 domain of the CFTR protein, which causes misfolding due to the p.Phe508del variant. Therefore, these compounds enable full maturation and membrane addressing of p.Phe508del CFTR, and preclinical studies have already demonstrated their efficacy in combination with a CFTR potentiator to restore CFTR activity close to wt level [134]. SION-719, SION-638 and SION-451 belong to this category of correctors targeting NBD1 (Table 1). Currently, a phase II CT is enrolling to evaluate SION-719 safety and tolerability in pwCF with two copies of p.Phe508del variants and already taking elexacaftor/tezacaftor/ivacaftor (NCT07108153). SION-451 also stabilizes NBD1 and is currently being evaluated in phase I CT in combination with galicaftor and SION-109 to evaluate its safety (NCT07035990). Finally, SION-638 was the first NBD1 stabilizer evaluated in a phase I CT in healthy volunteers (Table 1). For now, no further CT is planned with this molecule.

### 3.3. Other Types of Modulators

In addition to current potentiators and correctors, other CFTR modulators under development act as stabilizers, amplifiers, or read-through agents. The purpose of stabilizers is to increase CFTR stability at the PM (e.g., class IV variants), thus enhancing its half-life [16,135]. Nevertheless, this approach could be applied to many variants such as p.Phe508del, because of the reduced half-life of rescued p.Phe508del. Cavosonstat (N91115), an inhibitor of S-nitrosoglutathione reductase (GSNOR), increases GSNO activity and promotes mutated-CFTR stability at the PM (Table 1 and Figure 2) [136]. A phase I study conducted in healthy and pwCF homozygous for p.Phe508del demonstrated that cavosonstat is well tolerated. Moreover, a significant reduction in sweat Cl^−^ was observed at the highest dose [137]. However, a phase II study combining cavosonstat with ivacaftor and lumacaftor did not demonstrate any added pulmonary benefit. For now, no further trial is planned for this compound (NCT02589236). However, other stabilizers are under preclinical investigation. As an example, 1,2,4-thiadiazole is a CFTR stabilizer that aims to improve the p.Phe508del half-life by inhibiting its ubiquitination (Table 1). In fact, 1,2,4-thiadiazole derivative acts as an inhibitor of the ubiquitin ligase RNF5/RMA1 that is involved in early ubiquitination of p.Phe508del CFTR during its biosynthesis [138]. Thus, this molecule reduces ubiquitination of misfolded CFTR and increases its half-life [135,138]. Another stabilizer, iCAL36, a direct inhibitor of CFTR-associated ligand (CAL), is a PDZ domain-containing protein under preclinical development (Table 1). CAL is known for its ability to bind CFTR at the PM and promotes its recycling and lysosomal degradation [139,140]. Thus, iCAL36 aims to selectively inhibit CAL and CFTR protein interaction by competitively disrupting the CAL-CFTR interaction. This strategy demonstrated that iCAL36 improves p.Phe508del CFTR-dependent chloride efflux [140].

Amplifiers stabilize both wt- and p.Phe508del CFTR mRNA, thus increasing the amount of CFTR protein. Nesolicaftor (PTI-428) exemplifies this type of modulator as it increases CFTR protein levels in nasal mucosa by 50%, but it did not increase ppFEV_1_ in pwCF during the following CT (NCT03258424) [141,142]. However, a phase IIb CT is ongoing with a triple combination of nesolicaftor, dirocaftor (potentiator) and posenacaftor (corrector) (Table 1 and Figure 2) (NCT06468527).

Finally, read-through agents are molecules that promote the recruitment of another tRNA ‘to read’ a PTC (Figure 2), thus inducing the incorporation of a foreign amino acid and thereby enabling mRNA translation into a full-length CFTR protein [2,143]. These compounds are designed for class I variants, in which the presence of a PTC triggers the recruitment of the NMD machinery, which recognizes and degrades nonsense mRNA [50]. As an example, ELX-02 is a read-through agent that was evaluated in preclinical studies for its capacity to restore CFTR function in Gly542X (X: any other nucleotide) and Gly55X CFTR variants, and CFTR function can be further improved by modulators (Table 1) [144,145]. This compound was also evaluated in phase II CT with pwCF harboring at least one Gly542X allele (NCT04126473). This compound is no longer in clinical development but demonstrated proof of principle for this therapeutic strategy. Thus, other read-through agents such as SRI-41315 and CC-90009, which are eRF1 and eRF3a degraders, respectively, are in development for CF (Table 1) [146]. Both eRF1 and eRF3a are eukaryotic release factors, and their interaction with the ribosomal complex is critical for initiating NMD via the nonsense-mediated RNA decay [147,148]. Briefly, the degradation of eRF1 and eRF3a inhibits nonsense mRNA degradation. Recent work by Borelli and colleagues demonstrated that SRI-41315 and CC-90009 act differently regarding CFTR variants [146]. In fact, SRI-41315 was effective in restoring CFTR-dependent chloride efflux when associated with another NMD inhibitor in W1282X-CFTR but not in the Gly542X mutant, whereas CC-90009 was effective in rescuing the Gly542X mutant and less effective for Trp1282X [146].

Overall, marketed modulators are life-changing for pwCF, becoming the main cause of improvements in pwCF life expectancy. This is largely due to the diversity of CFTR modulators’ mechanisms of action that enable combination therapies, resulting in synergistic effects arising from the correction and potentiation of mutant CFTR proteins. Furthermore, in-development modulators suggest that new treatments could be marketable in the coming years, which would improve treatment efficacy and maybe eligibility. However, all these developed modulators have shown the scientific community that no unique molecule will be sufficiently efficient and will fit for all pwCF. Thus, combined therapeutic approaches are required.

**Table 1:** Overview and classification of approved and in-development modulators.

Modulator Class	Molecule	Type	Mechanism of Action	Development State
**Activator**	Genistein	/	Increase intracellular AMPc levels	Discontinued (NCT00590538)
**Potentiator**	Ivacaftor	/	Increase channel-opening probability via direct interaction with MSD2	Approved [85,149]
	Deutivacaftor	/		
	Dirocaftor	/	Increase channel-opening probability	Phase II CT (NCT06468527)
	VX-118	/		Phase I CT (NCT06154447)
	Sion-3067	/	Increase channel-opening probability via direct interaction with MSD2	Discontinued (NCT04853368)
	Icenticaftor	/	Increase channel-opening probability	Discontinued for CF (NCT02190604)
**Corrector**	Lumacaftor	Type I	Stabilize CFTR protein during its synthesis via direct interaction with MSD1	Approved [83,118]
	Tezacaftor			
	Galicaftor			Phase I CT (NCT07035990)
	Core-corr-II	Type II	Stabilize CFTR protein during its synthesis via direct interaction at the interface between NBD1 and NBD2	Discontinued [112]
	C4			
	GLPG2737		Unknown	Discontinued [129]
	Elexacaftor	Type III	Stabilize CFTR protein during its synthesis via direct interaction at the interface between TDM1 and MSD2	Approved [20,85]
	Vanzacaftor			
	Posenacaftor			Phase II CT (NCT06468527)
	IDOR-4	Type IV	Stabilize CFTR protein during its synthesis via direct interaction with MSD1	Preclinical studies [113]
	Sion-719	Undefined	Stabilize CFTR protein during its synthesis via direct interaction with NBD1	Phase II CT (NCT07108153)
	Sion-451			Phase I CT (NCT07035990)
	Sion-109		Stabilize CFTR protein during its synthesis via direct interaction at the interface between NBD1 and MSD2	
	Sion-638		Stabilize CFTR protein during its synthesis via direct interaction with NBD1	Discontinued
	VX-828	Undefined	Unknown	Phase I CT (NCT06154447)
**Amplifier**	Nesolicaftor	/	Stabilize CFTR mRNA thanks to the recruitment of the protein PCBP1	Phase II CT (NCT06468527)
**Stabilizer**	iCal36	/	Inhibit CFTR protein interaction with ligands involved in its plasma membrane recycling	Preclinical studies [140]
	1,2,4-thiadiazole derivative	/	Inhibit CFTR ubiquitination at the plasma membrane	Preclinical studies [138]
	Cavosonstat	/	Inhibit chaperones involved in CFTR plasma membrane recycling	Discontinued (NCT02589236)
**Read-through agents**	ELX-02	/	Enable the reading of PTC	Discontinued (NCT04126473)
	SRI-41315	/	Inhibit a component from the NMD pathway involved in CFTR mRNA degradation	Preclinical studies [146]
	CC-C90009	/		

### 3.4. Remaining Challenges with Approved Modulator-Based Treatments

Even though CFTR modulators have been life-changing for most patients, they remain insufficient to treat all pwCF. In fact, about 10% of Europe’s CF population are not eligible for current CFTR modulator therapies mainly due to their genotype, lung transplant status, or other factors, and still lack access to an effective treatment [150]. Moreover, this eligibility data is based on the European population and main variants, which differ in countries outside Europe such as Brazil and Turkey [151]. Even in Europe, these global data do not suggest that 90% of pwCF are indeed treated with CFTR modulators, and various data from national patients’ organizations highlight a gap between the number of eligible patients and the number of pwCF treated with approved CFTR modulators. As an example, the French association *Vaincre la Mucoviscidose* reported that in 2024, only 71.3% of the total French pwCF population was treated with CFTR modulators, while in Germany, this figure is near to 59.0%. Furthermore, in Germany, 83.2% and 64% of non-transplanted adults and children with CF, respectively, had a prescription for at least one CFTR modulator (data from the French cystic fibrosis registry, 2024, and the German cystic fibrosis registry, 2023). Globally, these figures are even lower, with estimates suggesting that as few as 27% of pwCF receive treatment with CFTR modulators, the current cost of CFTR modulators being the major reason for lack of access [3].

Despite improvements in ppFEV_1_, body mass index (BMI), sweat chloride concentration and pulmonary exacerbation rate, a significant proportion of pwCF treated with CFTR modulators experience multiple adverse events resulting from these therapies (Table 2). As reported in Table 2, these side effects are mainly headaches, diarrhea, upper respiratory tract infections and hepatotoxicity (based on European Medicines Agency data). Furthermore, recent studies highlighted that CFTR modulators induce, especially in women, anxiety and depression [152,153,154]. Indeed, many reports showed that some pwCF experienced mental health issues after starting the treatment and therefore required dose reduction or treatment discontinuation [121,152,153,155]. Studies investigating the impact of CFTR modulators on the mental health of pwCF are therefore necessary.

Moreover, pwCF already have a large medical burden due to symptomatic treatments, such as pancreatic enzyme replacement therapy, antibiotics, mucolytics and more, all associated with physiotherapies, and CFTR modulators administered at least once a day increase the medical burden [156,157,158,159,160]. However, recent studies indicate that the treatment burden could be reduced by discontinuing supportive therapies such as inhaled mucoactives agents in pwCF treated with elexacaftor/tezacaftor/ivacaftor [161,162]. In fact, reducing the treatment burden is a strong desire in the majority of pwCF [162].

Overall, modulator treatments have led to enormous progress in the treatment of CF. Currently, these therapies are available for young children aged 2 years old and older in many countries, leading to early treatment, which contributes to slowing disease progression. In fact, a recent CT performed with adolescents demonstrated that early access to modulators can reverse bronchial dilatation and reduce permanent airway damage [163]. Moreover, an ongoing CT aims to evaluate elexacaftor/tezacaftor/ivacaftor in children aged 12 to 23 months (NCT05882357) [164]. In the coming years, based on case reports and preclinical data from animal models, a CT evaluating the prenatal uses of CFTR modulators is expected to begin [165]. Thus, further data are still needed to clearly evaluate the benefit of access to modulators for newborns and to determine when these therapies can be safely initiated.

Nevertheless, the data discussed above highlights that treatment efficiency and eligibility are two criteria that still need to be improved. Therefore, other therapeutic approaches may help bridge this gap by expanding treatment options for pwCF who are ineligible for, unresponsive to, or intolerant of CFTR modulators, besides pwCF living in countries with no access to these treatments.

**Table 2:** CFTR modulator eligibility in Europe and observed benefits and side-effects (based on EMA data).

Molecule(s) and Common Dose	Relevant Variant(s)	Benefits	Most Common Adverse Effects
**Ivacaftor**	G551D, R117H, S1251N, Class IV and V variants	+10% FEV_1_ −55% pulmonary exacerbations −48 mmol/L sweat chloride +2.7 kg	Headache (23.9%) Oropharyngeal pain (22%) Upper respiratory tract infection (22%) Nasal congestion (20.2%) Abdominal pain (15.6%) Rhinopharyngitis (14.7%) Diarrhea (12.8%) Elevated transaminases (12.8%)
**Lumacaftor- ** **ivacaftor**	p.Phe508del homozygous	+2.6% FEV_1_ −30% pulmonary exacerbations −20.4 mmol/L sweat chloride +0.1 kg/m^2^ BMI	Dyspnea (14%) Diarrhea (11%) Nausea (10.2%)
**Tezacaftor- ** **ivacaftor**	p.Phe508del homozygous or p.Phe508del heterozygous with a class IV or V variant	+6.8% FEV_1_ −35% pulmonary exacerbations −10.1 mmol/L sweat chloride +0.06 kg/m^2^ BMI	Headache (14%) Rhinopharyngitis (12%)
**Elexacaftor- ** **tezacaftor- ** **ivacaftor**	At least one p.Phe508del variant	+10% FEV_1_ (p.Phe508del homozygous) and +13.8% FEV_1_ (p.Phe508del heterozygous with a functional variant) −63% pulmonary exacerbations −41 mmol/L sweat chloride (p.Phe508del homozygous) and −45 mmol/L sweat chloride (p.Phe508del heterozygous with a functional variant) +1.04 kg/m^2^ BMI	Headache (17.3%) Diarrhea (12.9%) Upper respiratory tract infection (11.9%)
**Vanzacaftor- ** **tezacaftor- ** **deutivacaftor**	At least one p.Phe508del variant	Same efficacy as elexacaftor plus tezacaftor plus ivacaftor	Headache Diarrhea Upper respiratory tract infection Nasopharyngitis Elevated transaminases

## 4. Targeting Non-CFTR Channels

An alternative variant-agnostic therapeutic approach for CF aims to bypass CFTR channel dysfunction by modulating alternative ion transport pathways in epithelial tissues [166]. One strategy focuses on activating other apical membrane Cl^−^ channels to restore anion flow and improve mucus clearance. A key target in this context is TMEM16A/anoctamin 1, a calcium-activated Cl^−^ channel. Its regulation and functional characteristics suggest that effective, long-acting small molecules capable of directly activating TMEM16A are needed to compensate for CFTR deficiency [166,167,168,169,170]. Recently, a study proposed to impact microRNA-9 function. The latter is involved in reducing TMEM16A expression and therefore diminishes Cl^−^ efflux by direct interaction with TMEM16A mRNA [171]. Thus, they developed ASOs targeting TMEM16A mRNA and competing with microRNA-9. Therefore, TMEM16A mRNA is less degraded, and protein expression is enhanced, restoring Cl^−^ efflux in multiple CF models [172]. However, the benefits of TMEM16A activation are controversial in the scientific community. Whereas many studies provide evidence of the need for TMEM16A expression and function, other studies demonstrated the TMEM16A implication for mucus secretion in airway and intestinal epithelial cells [173,174]. Based on these results, inhibiting TMEM16A expression could reduce mucus production. Thus, implications of TMEM16A activation and inhibition for CF still need to be elucidated.

Another promising target is SLC26A9, a member of the solute carrier 26 family, identified through genetic studies of lung disease. SLC26A9 plays a role in Cl^−^ transport and has the potential to support anion movement independently of CFTR, offering an alternative route to alleviate CF symptoms [166,170,175,176]. However, no SLC26A9 agonist molecule has been developed.

Additionally, another approach involves the use of artificial anion transporters (termed anionophores), i.e., small amphiphilic synthetic molecules designed to bind Cl^−^ and permit its diffusion across lipid bilayers, thus facilitating anionic transport across cell membranes [166]. Recent advances have led to the development of biologically active, non-toxic versions of these transporters, which could be applied to CF epithelia to restore ion balance. As an example, studies evaluated amphotericin B to improve HCO_3_^−^ secretion. In fact, this molecule forms unselective ion channels allowing HCO_3_^−^ secretion and thus increasing airway surface liquid pH [177,178]. Other products have the potential to increase Cl^−^ permeability of airway epithelia in the absence of functional CFTR channels [179]. However, ion transport through these molecules is not subject to regulation, which is a major drawback of this strategy.

Another complementary approach involves inhibiting ENaC, which becomes hyperactive in CF and contributes to airway dehydration; its inhibition can help restore airway surface liquid and improve mucociliary clearance [180,181,182,183,184]. Many CTs have taken place to reduce ENaC function. For example, even if inhaled ENaC inhibitor BI 1265162 was safe and well tolerated, it did not demonstrate any clinical benefit [185]. Moreover, the ongoing CT using the ENaC blocker ETD001 showed good safety, and a slight improvement (3.4%) in patients’ lung function was reported in early 2026 [180,186].

Together, these strategies represent a complementary or standalone alternative to current CFTR modulator therapies, particularly for individuals with variants unresponsive to existing drugs.

## 5. Theratyping vs. Theranostics

**Figure 4:**
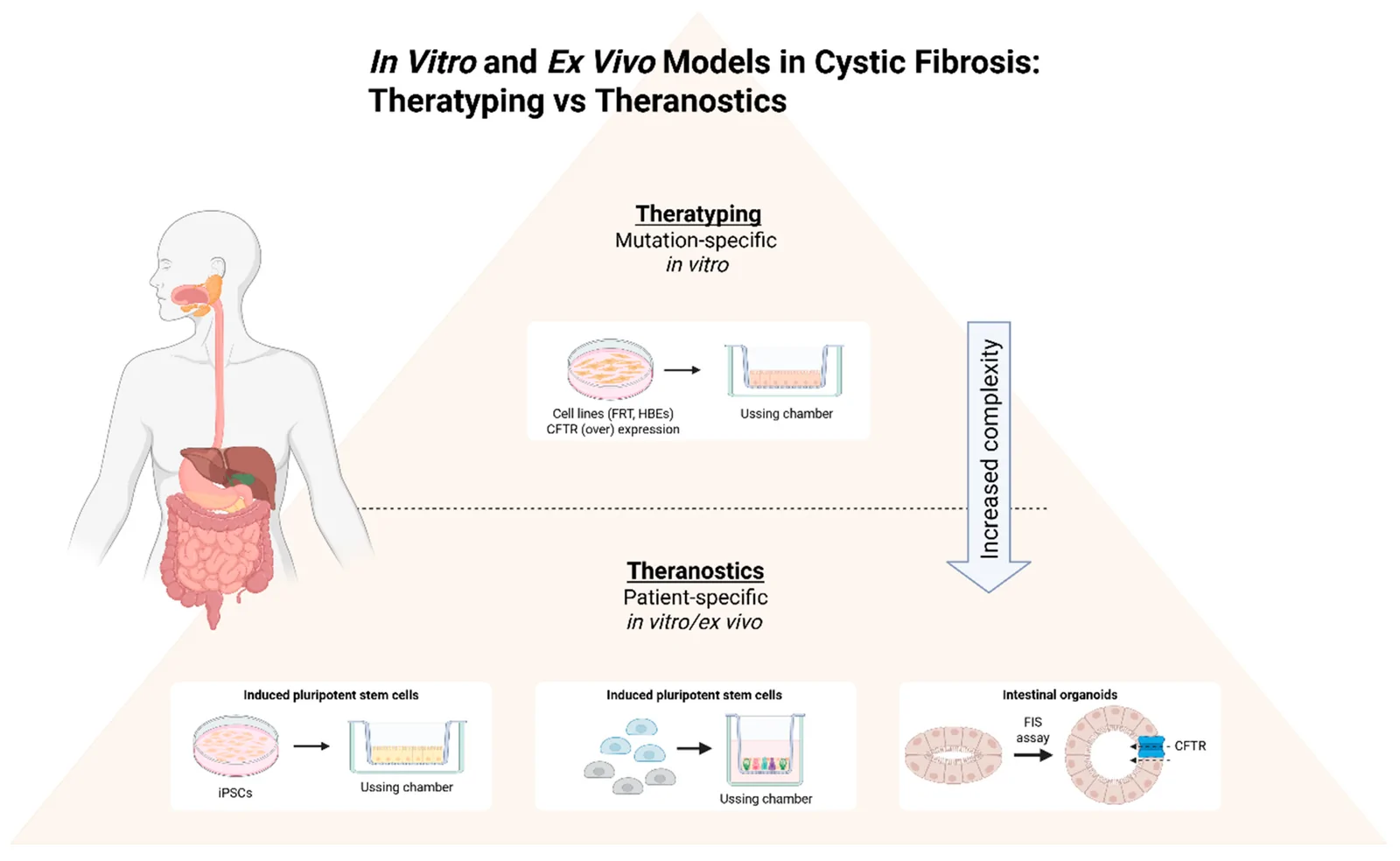
In vitro and ex vivo models in cystic fibrosis: theratyping vs. theranostics. Theratyping and theranostics are two approaches to appreciate the clinical benefit of the treatment being evaluated. While theratyping evaluates mutation-specific effects on established cell lines, which is an inexpensive approach, theranostics is more expensive and provides information on treatment efficacy from patient-derived mutant cells, thus considering in vivo complexities such as interindividual differences (created with Biorender.com).

‘Theranostics’, combining therapy and diagnostics, enables personalized treatment by predicting which drugs offer the best benefit to an individual. In CF, PDIOs and nasal epithelial cells (HNEs), in particular, were demonstrated to be physiologically relevant models to predict clinical responses to CFTR modulators (Figure 4) [187]. These systems reflect individual-specific genetic factors (not just in CFTR but also in modifier genes), epigenetic and phenotypic differences. They are good predictors of clinical outcomes by evidencing good correlations with parameters such as sweat Cl^−^ levels and lung function. Notwithstanding the recent EMA approval of CFTR modulators for all individuals with CF harboring at least one non-class I variant, rooted in the French compassionate program, PDIO/HNE assays remain an indispensable and validated preciSION-medicine tool to stratify responders and enable prompt therapy initiation in critically ill patients who have rare variants and cannot wait for national approval [188,189,190,191]. Their use helps prevent futile exposure and the associated psychosocial burden in non-responders, optimizes the allocation of healthcare resources, and ensures a robust in vitro–in vivo correlation that maximizes both clinical and pharmacoeconomic benefit [27,123,192,193,194,195].

Moreover, PDIOs allow for high-throughput drug screening and live-cell biobanking, supporting long-term testing and access to new treatments with no need to repeat biopsy. Centralized analysis of shipped samples is feasible and effective, especially for rare variants, where traditional trials are impractical. However, challenges remain, as generating and maintaining these models is technically demanding, resource-intensive, and dependent on access to pwCF samples in accredited centers. Though theranostics may over- or underestimate clinical benefits due to in vivo complexities (e.g., inflammation), they generally align well with real-world outcomes [194,196]. In contrast, cell line-based theratyping (Figure 4) is easier and more cost-effective for common variants but lacks patient specificity [197,198,199]. A combined approach—theratyping for common variants and theranostics for rare ones—may optimize treatment development and personalization across the CF population [196].

## 6. Combining Gene Therapy with Modulator Treatments: A Promising Area of Research

Several studies have demonstrated that CFTR modulators interact not only with mutant CFTR but also with wt-CFTR [86,87,88]. In fact, approved CFTR modulators have already shown preliminary effects on wt-CFTR protein levels and function [37,116,200]. Treatment of cells expressing wt-CFTR with lumacaftor, tezacaftor, and elexacaftor led to an increase in CFTR levels [116,200]. Similarly, a synergistic increase in wt-CFTR function (1.3–1.8-fold) was observed in rSIV/HN transduced HBE cells after the addition of ivacaftor [37]. Type IV correctors currently under development have also been shown to bind both mutant and wt-CFTR, increasing its abundance [113]. Moreover, amplifiers such as PTI-428 also act on wt-CFTR by stabilizing its mRNA and enhancing protein synthesis [141]. Furthermore, icenticaftor, initially evaluated for CF, was evaluated in CT for COPD. This compound was evaluated in CT in individuals expressing wt-CFTR and demonstrated a favorable safety and tolerability profile. However, as discussed above, modest improvements in lung function were reported with this molecule [107]. Along the same lines, a recent study compared the effects of ivacaftor and icenticaftor on CFTR function and showed that icenticaftor can improve wt-CFTR function [109]. Collectively, these findings suggest that CFTR modulators could potentiate the activity of wt-CFTR expressed following gene transfer, thereby providing additive or even synergistic therapeutic effects [88,98,107,109,115].

In addition, various studies have investigated nanoparticles as carriers for aerosol delivery of CFTR modulators such as ivacaftor and lumacaftor [201,202]. These proof-of-concept studies demonstrate that CFTR modulators can be efficiently encapsulated within liposomal nanoparticles for direct aerosol delivery to the lungs. Encapsulation improved CFTR-dependent chloride efflux compared with free ivacaftor and lumacaftor in CF cell lines [201]. In this context, ivacaftor has a better retention time in the lungs than free ivacaftor [202]. These data suggest that modulators can be efficiently encapsulated in nanoparticles for aerosol delivery. To date, for CF, no study has been conducted using nanoparticles loaded with both nucleic acids and modulators. However, combining gene transfer with small molecules has already been explored in other fields, suggesting the possibility of forming such nanoparticles for CF [203].

Theoretically, gene replacement approaches (using viral or non-viral vectors) and ASOs could lead to the expression of a wt-CFTR whose function could be enhanced using potentiators. Likewise, CFTR synthesis could be intensified using amplifiers, and CFTR protein folding could be improved with current correctors. Thus, the potency of each modulator needs to be investigated in association with gene transfer to determine if this combination could increase total CFTR amount and/or function.

To date, no CT evaluating CF gene transfer has required the discontinuation of CFTR modulators in participants already receiving these therapies [1]. Consequently, future gene transfer CT will mostly be conducted in combination with ongoing CFTR modulator therapy. This highlights that, for most patients, CFTR modulators will already be present at the time of gene therapy treatment, as illustrated by the CT using LNP-mediated CFTR mRNA delivery [45]. Thus, dedicated investigations are needed to elucidate whether modulators interfere with, have no impact on, or enhance the efficacy of gene therapy approaches.

Moreover, further research is needed to improve gene therapy strategies, particularly for airway delivery. This could be achieved by improving aerosol delivery techniques, developing vectors capable of penetrating thick mucus and specifically targeting airway epithelial cells, or carrying cell-specific promoters, and perhaps also by combining it with CFTR modulators [204]. Together, these data support the development of optimized nanoparticles that could be composed of both nucleic acids for gene delivery and modulators [201,202].

If combination therapy proves to be safe and provides additional clinical benefit, it could offer new opportunities to overcome various challenges of licensed treatments. For example, adverse events caused by CFTR modulators could be reduced by reducing the treatment dose. Immunogenicity induced by a combined therapeutic approach will also need to be investigated. These issues can be addressed using aerosolized synthetic vectors, which are less immunogenic than viral vectors, to bypass the hepatic first-pass effect and reduce hepatic toxicity. As an example, in the CT published in 2023 by Rowe et al., which aimed to deliver LNP loaded with CFTR mRNA, no severe immunogenicity or toxicity was observed in patients even if they were already taking CFTR modulators.

Finally, the overall treatment cost may be reduced by lowering modulator treatment doses, which represent a cost of approximately US$300,000 per patient per year in most countries [205]. This supposition may, however, depend on the gene transfer approach. As is known for current viral gene therapy in other diseases, manufacturing costs for these therapies are extremely expensive [206,207].

Overall, combining gene transfer strategies with CFTR modulators has the potential to improve treatment efficacy, expand eligibility, reduce the treatment burden, and possibly decrease treatment costs. Although this concept remains largely unexplored, the available preclinical evidence strongly supports further investigation of this therapeutic strategy.

## 7. Conclusions

CFTR modulator therapies have transformed the treatment of cystic fibrosis but remain insufficient to benefit all pwCF. In this review, we have highlighted cumulative evidence demonstrating that CFTR modulators can enhance not only mutant CFTR but also wt-CFTR expression and function. These findings are supported by extensive studies from multiple groups who have demonstrated the use of potentiators, correctors, and amplifiers to improve wt-CFTR protein levels and function. As suggested by Alton et al., we believe that, in the coming years, combining gene therapy with CFTR modulators represents a promising strategy that deserves to be strongly explored to improve pwCF treatments [39]. Such a combination has the potential to overcome the weaknesses of each approach while, together with complementary approaches such as theratyping and theranostics, it may bridge current therapeutic gaps to enable treatment for all pwCF.

## Data Availability

No new data were created or analyzed in this study. Data sharing is not applicable to this article.
